# Design and Implementation of a Novel Web-Based E-Learning Tool for Education of Health Professionals on the Antibiotic Vancomycin

**DOI:** 10.2196/jmir.6971

**Published:** 2017-03-30

**Authors:** Stuart Evan Bond, Shelley P Crowther, Suman Adhikari, Adriana J Chubaty, Ping Yu, Jay P Borchard, Craig Steven Boutlis, Wilfred Winston Yeo, Spiros Miyakis

**Affiliations:** ^1^ Wollongong Hospital Department of Pharmacy Illawarra Shoalhaven Local Health District Wollongong Australia; ^2^ School of Medicine Faculty of Science, Medicine and Health University of Wollongong Wollongong Australia; ^3^ Illawarra Health and Medical Research Institute University of Wollongong Wollongong Australia; ^4^ St George Hospital Department of Pharmacy South Eastern Sydney Local Health District Kogarah Australia; ^5^ St George Clinical School Faculty of Medicine University of New South Wales Kogarah Australia; ^6^ Prince of Wales Hospital Department of Pharmacy South Eastern Sydney Local Health District Randwick Australia; ^7^ School of Computing and Information Technology Faculty of Engineering and Information Sciences University of Wollongong Wollongong Australia; ^8^ Research Central Wollongong Hospital Illawarra Shoalhaven Local Health District Wollongong Australia; ^9^ Wollongong Hospital Department of Infectious Diseases Illawarra Shoalhaven Local Health District Wollongong Australia; ^10^ Wollongong Hospital Division of Medicine Illawarra Shoalhaven Local Health District Wollongong Australia

**Keywords:** nursing education, pharmacy education, medical education, continuing education, vancomycin, survey methods, anti-bacterial agents

## Abstract

**Background:**

Traditional approaches to health professional education are being challenged by increased clinical demands and decreased available time. Web-based e-learning tools offer a convenient and effective method of delivering education, particularly across multiple health care facilities. The effectiveness of this model for health professional education needs to be explored in context.

**Objectives:**

The study aimed to (1) determine health professionals’ experience and knowledge of clinical use of vancomycin, an antibiotic used for treatment of serious infections caused by methicillin-resistant *Staphylococcus aureus* (MRSA) and (2) describe the design and implementation of a Web-based e-learning tool created to improve knowledge in this area.

**Methods:**

We conducted a study on the design and implementation of a video-enhanced, Web-based e-learning tool between April 2014 and January 2016. A Web-based survey was developed to determine prior experience and knowledge of vancomycin use among nurses, doctors, and pharmacists. The Vancomycin Interactive (VI) involved a series of video clips interspersed with question and answer scenarios, where a correct response allowed for progression. Dramatic tension and humor were used as tools to engage users. Health professionals’ knowledge of clinical vancomycin use was obtained from website data; qualitative participant feedback was also collected.

**Results:**

From the 577 knowledge survey responses, pharmacists (n=70) answered the greatest number of questions correctly (median score 4/5), followed by doctors (n=271; 3/5) and nurses (n=236; 2/5; *P*<.001). Survey questions on target trough concentration (75.0%, 433/577) and rate of administration (64.9%, 375/577) were answered most correctly, followed by timing of first level (49%, 283/577), maintenance dose (41.9%, 242/577), and loading dose (38.0%, 219/577). Self-reported “very” and “reasonably” experienced health professionals were also more likely to achieve correct responses. The VI was completed by 163 participants during the study period. The rate of correctly answered VI questions on first attempt was 65% for nurses (n=63), 68% for doctors (n=86), and 82% for pharmacists (n=14; *P*<.001), reflecting a similar pattern to the knowledge survey. Knowledge gaps were identified for loading dose (39.2% correct on first attempt; 64/163), timing of first trough level (50.3%, 82/163), and subsequent trough levels (47.9%, 78/163). Of the 163 participants, we received qualitative user feedback from 51 participants following completion of the VI. Feedback was predominantly positive with themes of “entertaining,” “engaging,” and “fun” identified; however, there were some technical issues identified relating to accessibility from different operating systems and browsers.

**Conclusions:**

A novel Web-based e-learning tool was successfully developed combining game design principles and humor to improve user engagement. Knowledge gaps were identified that allowed for targeting of future education strategies. The VI provides an innovative model for delivering Web-based education to busy health professionals in different locations.

## Introduction

### Internet-Based Learning

The development of Internet-based learning (IBL) for health care professionals has increased in recent years [1]. One reason for advancement of IBL is the existence of barriers associated with implementation of face-to-face health professional education, including increased clinical demands and decreased available time [2]. These barriers become more evident where education is required across multiple facilities that are separated by long distances. Consequently, there is a requirement for more effective and accessible ways of improving knowledge and competence in health professionals [1]. To date, IBL approaches have shown positive effects on health education outcomes through overcoming the above barriers [3].

Serious games have been defined as “interactive computer applications, with or without significant hardware components” that are designed to entertain while achieving changes in knowledge or skills [4,5]. Methods to improve their entertainment value include dramatic tension, humor, and challenge [5]. User engagement can also be improved through the inclusion of a narrative [6]. Humor as an aid to nursing and medical education has been described in the literature [7,8], where the use of games as a medium for humor may increase learners’ interest and motivation to learn [7]. As distinct from e-learning with limited user interaction [9], serious games can provide greater engagement with the educational content.

Use of serious game methodologies to deliver health professional education has been reported in previous studies [10-12]. Educational and design frameworks are recommended for the development of games for health professional education [9,13]. Strategies include application of knowledge in a safe environment that resembles real life [13], a degree of interactivity [14], and entertainment [5,9]. These topics were considered in development and assessment of the e-learning tool in this study.

Most e-learning tools in health care have targeted specific groups, such as medical or nursing students, physicians, or nurses [15-18]. We developed the Vancomycin Interactive (VI) to target nurses, doctors, and pharmacists, the three main groups involved in use of medicines in hospitals. The specific educational content of the VI was clinical use of the glycopeptide antibiotic, vancomycin, given intravenously in hospitals for treatment of infections caused by methicillin-resistant *Staphylococcus aureus* (MRSA). MRSA infections have high mortality and are resistant to conventional treatment with safer antibiotics such as penicillins, which usually do not require such specific administration and monitoring. Vancomycin is a commonly used antibiotic for treatment of MRSA infections [19], but there are problems associated with its use. Those include the requirement for a loading dose (initial higher single dose) in serious infections, side effects when administered too rapidly, and the need to monitor vancomycin plasma levels [20]. As part of our antimicrobial stewardship program [21], local quality improvement activities identified gaps in competence around clinical use of vancomycin. Three main topics were identified from those local activities and from previous studies: (1) dosing, including loading and maintenance [22-24]; (2) administration, such as compatible fluids and rate of infusion [24,25]; and (3) therapeutic drug monitoring (TDM), including appropriate timing of blood sampling, target trough levels, and required actions based on reported levels [24,26,27].

### Aims of This Study

The aims of this study were to (1) report the design and implementation of a Web-based, interactive e-learning tool providing education on the dosing, administration, and TDM of vancomycin, (2) assess health professionals’ preintervention knowledge of vancomycin use in order to inform development of the e-learning tool, and (3) assess health professionals’ initial acceptance of the VI.

## Methods

### Setting

**Figure 1:**
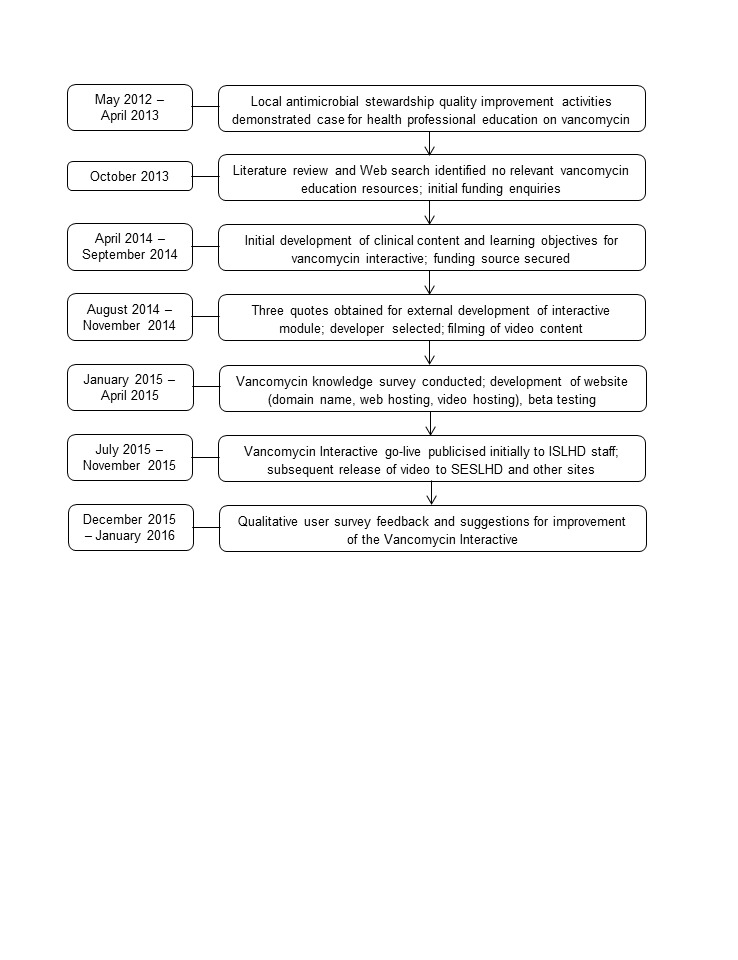
Timeline of Vancomycin Interactive design, implementation, and evaluation. ISLHD: Illawarra Shoalhaven Local Health District; SESLHD: South Eastern Sydney Local Health District.

This prospective design and implementation study of a video-enhanced, Web-based e-learning tool took place in Illawarra Shoalhaven Local Health District (ISLHD) and South Eastern Sydney Local Health District (SESLHD), located in New South Wales (NSW), Australia. These health districts cover a geographic area of 6331 km^2^ and have an estimated population of 1.17 million, reaching from central Sydney to 3 h drive south [28]. The districts’ 14 hospitals contain a total of 2500 beds and range from small rural facilities to large tertiary metropolitan hospitals. A timeline of design, implementation, and evaluation is shown in Figure 1.

### Web-Based Vancomycin Knowledge Survey

An anonymous open Web-based survey was created using SurveyMonkey (SurveyMonkey Inc, Palo Alto, CA) to determine confidence, experience, and knowledge of vancomycin before the VI. The survey was developed locally by the antimicrobial stewardship and educator pharmacists as part of routine activities, with input from the infectious diseases team. Clinical content was based on the Australian Therapeutic Guidelines: Antibiotic, Version 15, 2014 [20] and the Australian Injectable Drugs Handbook, Version 6, 2015 [29]. Use of these references was required as part of the Australian hospital accreditation standards [30]. Survey participants were nurses, doctors, and pharmacists from the two health districts. A 4-point Likert scale was used to determine levels of experience, confidence and knowledge on dosing, administration, and TDM of vancomycin (see Multimedia Appendix 1). The survey was advertised using email and the districts’ fortnightly newsletters. The survey link was open from February 1, 2015 to June 30, 2015, and participation was voluntary. Only one attempt was allowed on each question and users were directed to further reading material at completion of the survey. Nurses were expected to correctly answer questions on fluid compatibility and administration rate, since they were mainly responsible for administration of medicines in hospitals. Doctors were anticipated to correctly answer questions relating to dosing and TDM, arising from their role as prescribers. Pharmacists were expected to have a working knowledge of all aspects of clinical vancomycin use. The response rate to the survey was calculated from the number of respondents and the number of recipients on staff email groups.

### Design and Implementation of the Vancomycin Interactive

Similar to the survey, clinical content of the VI was developed locally, based on the Therapeutic Guidelines: Antibiotic [20] and the Australian Injectable Drugs Handbook [29]. The vancomycin knowledge survey informed in part the VI’s educational content in the postproduction phase, allowing finalization of the multiple choice questions. An entertaining Web-based educational tool was selected in the early development stage (mid-2013; Figure 1) for two reasons. First, there was already a health district requirement for staff to complete between 10 and 20 h mandatory training per year on other topics, and study investigators did not wish to contribute to the burden of further Web-based mandatory training. Rather, a brief, targeted, and light-hearted educational tool was thought to be more acceptable and beneficial for staff. Second, large distances between hospital sites meant that face-to-face education of health professionals was very resource intensive. The learning objectives of the VI for target users (nurses, doctors, and pharmacists) were to improve knowledge of vancomycin dosing, administration, and TDM. The VI did not address clinical indications for vancomycin, dosing in specialist areas such as intensive care and renal dialysis, use of continuous infusions, or surgical prophylaxis.

**Figure 2:**
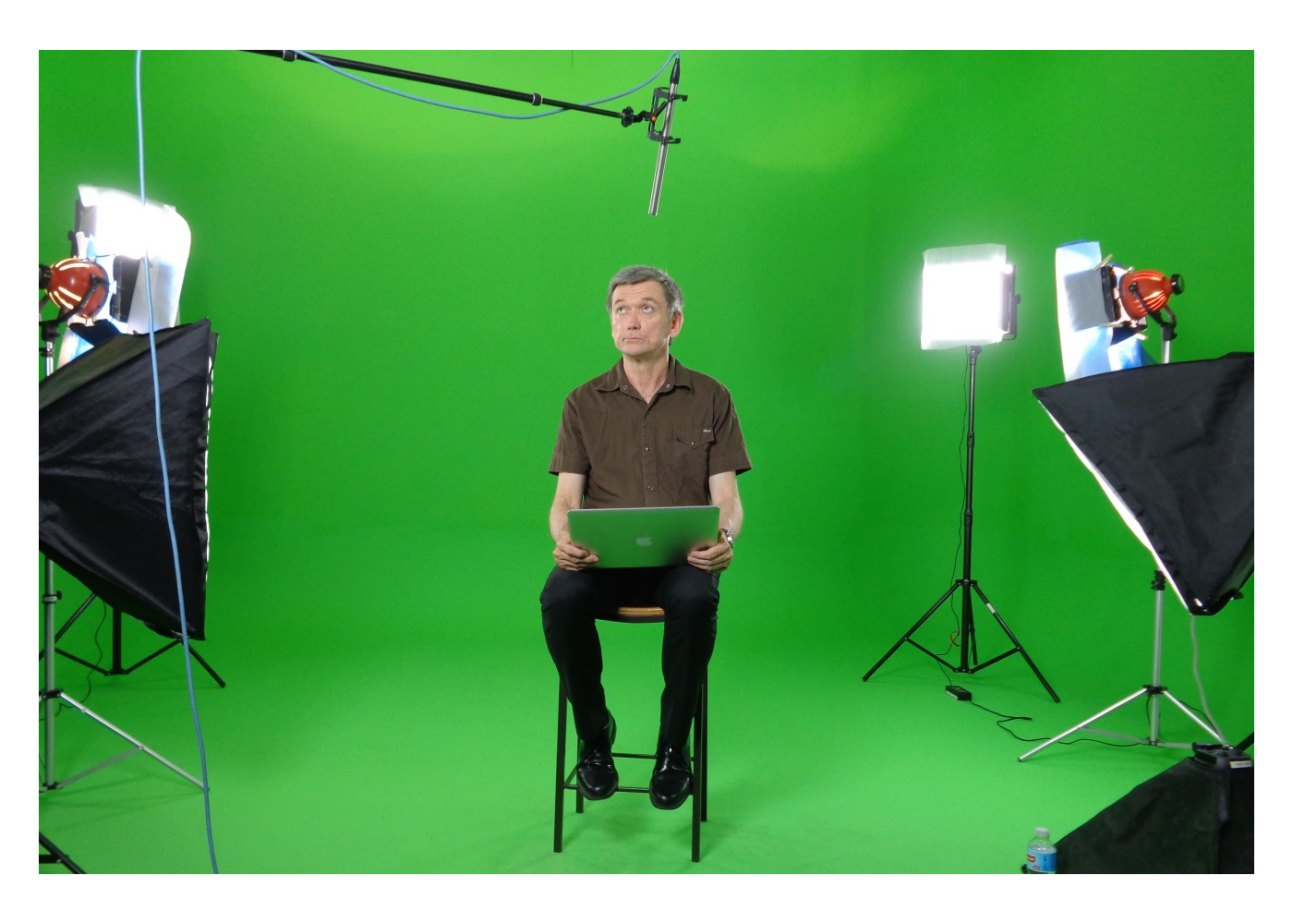
Filming of the Vancomycin Interactive (VI).

**Figure 3:**
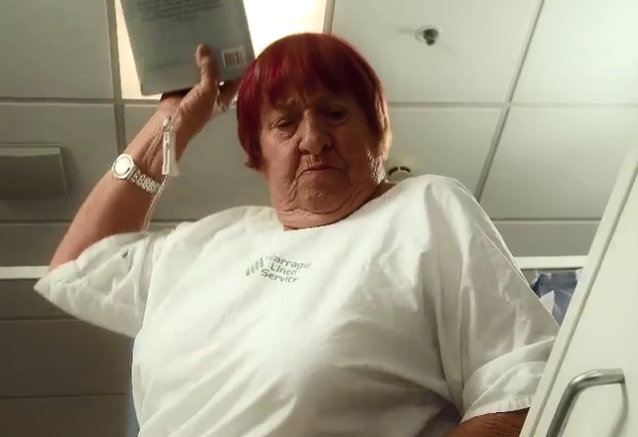
Dramatic tension created the basis for the Vancomycin Interactive’s plot.

A single interactive video was produced due to financial constraints; there was the expectation that all professional groups should have rudimentary knowledge of clinical vancomycin use. The VI (ISLHD) was hosted on an open website [31]. Using the serious game design concepts of interactivity and entertainment, we presented a case study resembling real-life interaction between a patient and a health professional. Dramatic tension between the two characters created the basis for the plot, along with the unprofessional behavior of the modeled health professional. The interaction was also designed to be humorous, particularly through the special effect of “shrinking” the health professional, and references to William Shakespeare’s plays (Figures 2 and 3). The concept was intended to appeal to health professionals who may feel that they are at the mercy of their patients, a theme that emerged during the script-writing process.

**Figure 4:**
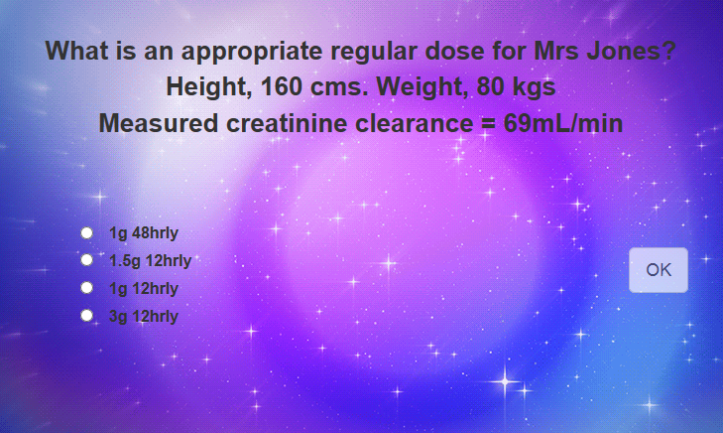
Example of user interface for an interactive question from the Vancomycin Interactive (VI).

The user interface consisted of video clips interspersed with interactive question and answer scenarios placed at the specific points, so that technical content felt organic to the narrative (Figure 4; Multimedia Appendix 2). A correct answer allowed progression to the next section, whereas an incorrect answer resulted in a shaking screen and a sound effect. Data captured from answers to the interactive questions allowed for subsequent analysis. Only data from the targeted health professionals were included in the analysis; students and other participants were excluded. Additional questions in the VI, as distinct from the survey, related to compatibility of vancomycin with various fluids and clinical actions in response to different trough levels. Completion of the VI took approximately 10 min based on user testing.

Quotes for production were obtained from three developers in accordance with NSW Health policy, with financial support provided internally by the Clinical Governance Unit of the health district. Content development began in April 2014, and the video was filmed using professional actors in November 2014. Postproduction modifications were made to the video until release in July 2015. In early 2015, the website was established to promote improved access to the VI, and to include additional clinical content not contained in the VI. Testing of content and usability was performed by pharmacists and infectious diseases doctors (N=8) at the study site, with feedback provided by email to the study investigators. Feedback from testers predominantly related to accuracy of the clinical content in the context of the narrative, and informed the final iteration of the VI. The first phase of dissemination and advertisement (email, newsletters, link on intranet home page) to ISLHD staff occurred on July 27, 2015 (Figure 1), with the initial target audience estimated from organizational records to be 1000 staff. General release of the VI outside of ISLHD occurred on November 17, 2015. The final production cost was Aus $15,000; time devoted to content development, testing, advertising, implementation, and evaluation was not included in those costs as it fell within usual activities for the pharmacy and infectious diseases department staff members involved in development of the VI.

### User Acceptance Evaluation

Following release of the VI, qualitative survey responses were assessed to inform the investigators about user acceptability and suggestions for improvement. The qualitative survey was open between December 1, 2015 and January 31, 2016, in order to conclude before the annual intake of new junior doctors in February 2016 (Figure 1).

### Outcome Measures

The primary outcome measure was comparative vancomycin knowledge between health professions and self-reported levels of confidence and experience. Vancomycin knowledge responses from website data (not linked at a participant level) were also assessed and compared with the knowledge survey. In addition, qualitative feedback on the VI was evaluated using a 5-point Likert scale and free text responses that were grouped into key themes. Assessments were derived from survey responses and VI website data. Technical issues around compatibility with desktop and mobile operating systems and Web browsers were also assessed. Reporting of outcomes on quantitative postintervention survey data, clinical measures of quality vancomycin use such as therapeutic vancomycin plasma levels, and clinical outcomes related to vancomycin treatment was beyond the scope of this study.

### Statistical Analyses

Chi-square and Fisher exact tests were used for proportions. Chi-square for trend was used to determine trend between professions for knowledge questions. Kruskal-Wallis and Mann-Whitney *U* tests were used to examine total survey scores. For continuous data, normality was assessed using the Shapiro-Wilk statistic. A skewed distribution was denoted by *P*<.05. Kruskal-Wallis and follow-up Wilcoxon rank-sum tests were used to investigate between subjects effects with nonnormal distributions. A multivariate analysis was performed to examine influential factors on correct survey responses. For each item, a logistic regression was conducted followed by a multiple regression on the total score. For profession, nurses were allocated to the reference group, and self-reported “no experience or confidence” was used at the reference for the experience analysis. Statistical significance was accepted as *P*<.05. Additionally, a mediation analysis [32] was carried out to explore the mediating effects of vancomycin experience on the association between profession and knowledge (reflected by the total number of correct responses). For the mediation analysis, significance was determined by the 95% CI of the regression coefficient, *b*. If the 95% CI did not contain 0, it was considered significant. The extent of mediation was reported as a percentage, where a higher percentage reflects greater mediation. Statistical analyses were performed using Stata statistical software: Release 14 (Statacorp LP).

### Ethics

Ethics approval was granted by the Joint University of Wollongong and Illawarra Shoalhaven Local Health District Health and Medical Human Research Ethics Committee (EC00150; approval number HE15/005). The VI website contained a disclaimer that anonymous data collected from the video could be used for research purposes.

## Results

### Vancomycin Knowledge Survey Before Release of the Vancomycin Interactive

The response rate to the survey was 26.87% (577 responses from 2147 email recipients). The response rates by profession were 24.4% (236/967) for nurses, 25.33% (271/1070) for doctors, and 63.6% (70/110) for pharmacists (*P*<.001).

As shown in Table 1, the median knowledge survey score for nurses was 2/5 (interquartile range, IQR 1-3), compared with 3/5 (IQR 3-4) for doctors and 4/5 (IQR 3-4) for pharmacists (*P*<.001). Pharmacists had greater total scores than doctors (*P*<.001) and nurses (*P*<.001), whereas doctors had greater total scores than nurses (*P*<.001). For nurses, the most correctly answered questions were on administration rate (64.4% correct; 152/236) and target trough range (57.6% correct; 136/236), whereas only 19.5% (46/236) of nurses answered the loading dose question correctly. The most correctly answered question by doctors was on target trough range (86.3% correct; 234/271). Pharmacists answered all responses correctly greater than 80% of the time.

**Table 1:** Number of correct responses to Web-based vancomycin knowledge survey.

Survey question	Nurse (n=236) n (%)	Doctor (n=271) n (%)	Pharmacist (n=70) n (%)	*P* value	Total (N=577) n (%)
Loading dose	46 (19.5)	112 (41.3)	59 (84)	<.001	217 (37.6)
Maintenance dose	58 (24.6)	126 (46.5)	58 (83)	<.001	242 (41.9)
Administration rate	152 (64.4)	160 (59.0)	62 (89)	<.001	374 (64.8)
First level timing	70 (29.7)	155 (57.2)	59 (84)	<.001	284 (49.2)
Target trough range	136 (57.6)	234 (86.3)	65 (93)	<.001	435 (75.4)
Median total score (IQR^a^)	2 (1-3)	3 (3-4)	4 (3-4)	<.001	3 (2-4)

^a^IQR: interquartile range.

Multivariate analysis (Table 2) showed that for the loading dose question, pharmacists and doctors were more likely to achieve a correct response than nurses. A smaller variation between professions was seen for the administration rate question, with the comparison between pharmacists and nurses reaching significance. In addition, self-reported “very” and “reasonably” experienced health professionals were more likely to achieve a correct response. Similar associations between professions and experience levels were seen for maintenance dose, first level timing, and trough level range (Table 2). Pharmacists self-reported more experience and confidence than doctors or nurses, which influenced the likelihood of a correct response.

**Table 2:** Multivariate analysis of vancomycin knowledge survey responses (N=577).

Topic		Profession				Experience or confidence^a^			
		Nurse ref^b^	Doctor	Pharmacist	1 ref^b^		2	3	4
**Loading dose**	OR^c^ (95% CI)	-	2.6 (1.7-4.1)	16.8 (7.9-35.7)	-		1.4 (0.8-2.7)	4.6 (2.4-8.6)	11.1 (3.3-36.9)
	*P* value	-	<.001	<.001	-		.25	<.001	<.001
**Maint dose^d^**	OR (95% CI)	-	2.5 (1.7-3.8)	12.1 (5.9-24.7)	-		1.0 (0.6-1.8)	2.2 (1.2-3.8)	3.3 (1.1-9.6)
	*P* value	-	<.001	<.001	-		.85	.006	.03
**Admin rate^e^**	OR (95% CI)	-	1.0 (0.6-1.4)	2.9 (1.3-6.4)	-		2.1 (1.4-3.3)	4.7 (2.9-7.6)	5.7 (0.2-1.1)
	*P* value	-	.82	.01	-		.001	<.001	<.001
**Level timing^f^**	OR (95% CI)	-	2.7 (1.9-4.1)	8.5 (4.0-17.7)	-		3.6 (2.1-5.9)	6.8 (4.0-11.6)	4.0 (1.4-10.9)
	*P* value	-	<.001	<.001	-		<.001	<.001	.008
**Trough range^g^**	OR (95% CI)	-	3.9 (2.5-6.1)	5.6 (2.1-15.1)	-		3.4 (2.1-5.6)	5.7 (3.2-10.0)	5.7 (1.2-26.5)
	*P* value	-	<.001	.001	-		<.001	<.001	.03
**Total correct^h^**	*b*^i^ (95% CI)	-	.8 (0.6-1.1)	1.7 (1.4-2.1)	-		.9^j^ (1.4-2.1)		
	*P* value	-	<.001	<.001	-		<.001		

^a^Experience or confidence: 1, none; 2, a little; 3, moderate; 4, very experienced or confident.

^b^ref: reference group for multivariate analysis.

^c^OR: odds ratio.

^d^maint dose: maintenance dose.

^e^admin rate: administration rate.

^f^level timing: timing of first level.

^g^trough range: target range for plasma trough level.

^h^total correct: nurse (all levels), doctor (all levels), pharmacist (all levels).

^i^*b*: regression coefficient.

^j^Average of responses to three vancomycin experience or confidence questions, therefore a multiple regression was performed for total correct.

Subsequent mediation analysis revealed that vancomycin experience significantly mediated the effect of profession on total score (total indirect effect: *b*=.63, bias-corrected 95% CI 0.44-0.85). Approximately 58% of the profession effect was mediated by experience, where a higher percentage value indicates greater mediation.

### Vancomycin Interactive

Responses to the VI were analyzed using background website data received from July 27, 2015 to November 14, 2015, with ISLHD as the target population group. The initial dropdown question asking the user’s profession was answered by 389 participants, of which 163 health professionals (41.9% of those answering the initial profession question) completed all 10 questions (Table 3). The rate of correctly answered questions on first attempt was 65% for nurses, 68% for doctors, and 82% for pharmacists, significantly higher in the pharmacist group (*P*<.001). Notably low numbers of correct responses were identified for the following three questions, averaged over the three professional groups: loading dose (39% correct), timing of first level (50%), and timing of subsequent levels (48%).

**Table 3:** Number (%) of correct answers on first attempt by nurses, doctors, and pharmacists from VI data.

Question^a^	Nurse n=63	Doctor n=86	Pharmacist n=14	*P* value^b^	Total N=163
1. Loading dose	19 (30)	36 (42)	9 (64)	.05	64 (39)
2. Maintenance dose	50 (79)	59 (69)	11 (79)	.32	120 (74)
3. Compatible fluids	53 (84)	67 (78)	11 (79)	.76	131 (80)
4. Administration rate	56 (89)	55 (64)	14 (100)	<.001	125 (77)
5. Timing of first level	20 (32)	49 (57)	12 (86)	<.001	81 (50)
6. Target trough level	47 (75)	72 (84)	12 (86)	.34	131 (80)
7. Level of 35 mg/L	43 (68)	68 (79)	14 (100)	.02	125 (77)
8. Level of 20 mg/L	49 (78)	81 (94)	13 (93)	.01	143 (88)
9. Level of 26 mg/L	46 (73)	55 (64)	12 (86)	.20	113 (69)
10. Subsequent levels	27 (43)	45 (52)	7 (50)	.52	79 (48)
Average score	65%	68%	82%	<.001	68%

^a^VI questions are shown in Multimedia Appendix 2.

^b^*P* values obtained using chi-square for trend.

### Comparison of Responses Between Vancomycin Interactive (VI) and Web-Based Survey

The rates of correct response from the VI were significantly higher than the knowledge survey for maintenance dose (74% VI vs 42% survey; *P*<.001) and administration rate questions (77% VI vs 65% survey; *P*=.004). There was a slightly higher correct response rate for the question on target trough level (80% VI vs 75% survey; *P*=.19). Uniformly low correct response rates were observed for the questions on loading dose (39% for VI vs 38% for survey; *P*=.70) and the timing of first level (50% VI vs 49% survey; *P*=.89). The question on timing of levels subsequent to the first level in the VI was answered correctly in 48% of cases; there was no equivalent question in the survey.

### User Acceptance Evaluation of the Vancomycin Interactive

Among the 163 VI participants, 51 (31%) responses were received. Responses were predominantly positive, as shown in Table 4.

**Table 4:** Qualitative responses (%) following participation in the Vancomycin Interactive (VI).

Survey statement or question	Strongly agree	Agree	Neutral	Disagree	Strongly disagree
**Using the VI^a^ has enhanced my knowledge (n=51)**	11 (22)	29 (57)	8 (16)	3 (6)	0 (0)
Using the VI has improved my performance (n=50)	8 (16)	28 (56)	12 (24)	2 (4)	0 (0)

^a^VI: Vancomycin Interactive.

When users were asked, “What’s good about the VI in comparison to other e-learning modules?” 28 free text responses were received. It was found that 4 responses (14%) were related to not being able to load the video. Key themes from the remaining 24 responses (86%) were “entertaining,” “engaging,” “a lighter approach to learning,” “more real life,” and “held attention.” The question, “Does the training provided by the VI meet your needs? If not, what can be improved?” received 23 free text responses. A total of 16 respondents (70%) reported, “yes it met needs;” 2 users (10%) stated issues loading VI; 3 users (13%) requested printable resources; 1 user was “not sure;” and 1 user requested more information to be available when answering questions. All qualitative survey responses are provided in Multimedia Appendix 3.

## Discussion

### Principal Findings

We have reported on the design, implementation, and user evaluation of a novel Web-based e-learning tool for education of health professionals on clinical use of the antibiotic vancomycin. The VI was developed for noncommercial use and targeted three health professional groups across multiple hospital sites. Responses from the survey that preceded the VI demonstrated a global lack of knowledge on the safe and effective use of vancomycin among nurses and doctors, justifying a Web-based learning approach that was suitable for disparate geographical locations. Pharmacists were shown to be more knowledgeable on clinical vancomycin dosing, administration, and TDM.

As expected, self-reported levels of confidence and experience were correlated with increased likelihood of correct responses to the knowledge survey questions. Responses from the Web-based knowledge survey and VI data were only similar for three of the five common questions, loading dose, timing of first level, and the target trough level (see Multimedia Appendices 1 and 2). This may suggest that respondents equally understood those three questions in the VI and the knowledge survey. Responses to two questions, maintenance dose and administration rate, were significantly better in the knowledge survey compared with the VI. This could be caused by the respondents’ different understanding about the survey questions presented in the two media or difference in knowledge level between the participants in the two surveys. Following implementation, qualitative survey responses demonstrated that the VI was well-received by users, and was considered to be an engaging and entertaining method of improving knowledge. A small number of responses highlighted technical issues relating to not being able to load the video content, which were generally resolved through software upgrades.

Numerous studies have reported the development and evaluation of serious games for training health professionals, but few have targeted multiple professions [11,33-35]. One study reported development of a serious game on appropriate antibiotic use, but this was not specific to any particular antibiotic [12]. Vancomycin was chosen as the topic for our Web-based tool due to its frequency of use, and complexities associated with treatment of serious MRSA infections, the requirement for loading doses, TDM, and subsequent dose adjustment. The VI in this study adopted some principles of serious game design [9], including interactivity and entertainment, and combined those with humor [8] to engage multiprofessional users. Knowledge responses from the VI are promising, and further research is needed to determine the reasons for difference in responses to questions between the classical knowledge survey and knowledge responses from VI website data.

Feedback from the majority of the participants suggested that the VI enhanced their vancomycin knowledge (79%) and improved their performance (72%). This supports the VI as a Web-based resource to provide health care professionals with training on clinical use of vancomycin. Qualitative responses were generally positive, further supporting the use of the VI for health professional education. The main challenges for implementation of the VI related to developing clinical content for the video that would remain applicable to all three professional groups, without creating a tool that would take too long to complete. Advertising the tool using different media was also challenging, as the tool was made available across two health districts with multiple hospitals, and the target professional groups may have preferred to receive alerts regarding content in different ways.

The creation of a brief, Web-based, entertaining educational tool was the purpose of the project, whereby no further mandatory training burden was placed on staff. As distinct from existing local mandatory learning modules, the VI was intended for use among clinical staff involved in vancomycin use. Employing serious game design concepts may provide greater educational benefit than traditional computer-based learning methods through the use of greater interactivity, entertainment, and scoring; however, further published comparisons are required [11]. Our results suggest that pharmacists have the greatest level of knowledge on clinical vancomycin use. Therefore, to deliver the best learning outcomes for health professionals in this area, it is recommended to combine face-to-face teaching with VI learning, using pharmacists as educators in the future.

### Limitations of This Study

We studied the logistics and design of a Web-based e-learning tool incorporating interactive video content for health professional education relating to clinical use of the antibiotic vancomycin. Postintervention knowledge and clinical outcomes were not reported here; these form the basis of ongoing research that will be reported separately. The use of an open Internet site allowed for potential diffusion worldwide, since users outside our organizations may have found the VI using an Internet search engine. In August 2015, the website was also shared on a professional network with members outside the targeted health district. As a result, there was some unintended use of the video before its general release. However, the greatest number of Web sessions was from ISLHD, and employees of the target ISLHD hospitals may not have been physically located in the region while completing the VI.

Question design within the VI was limited to multiple choice and multiple answer questions. Further variation in question types such as open questions, as previously reported [16], could be made in future versions to improve immersion and interactivity. The inclusion of a formal testing process immediately before and after the e-learning tool may also have added some informative value on its effect and could inform future improvements. In addition, further scoring methodology, such as time limitation, competition, and increasing difficulty could improve the robustness of the design [11]. The Hawthorne effect may have introduced bias into the study, whereby participants’ behavior may have been altered through their awareness of being measured. This bias may have been limited by participation being anonymous, and the primary intended aim for users being to further their vancomycin knowledge, rather than participation in a research project. Detailed economic analysis of the study was limited by the project forming part of usual educational activities for study investigators. As such, the total project cost was likely greater than the reported production cost.

There was relatively low uptake of the VI among clinical staff during the study period despite broad advertisement; this limited the statistical power of the study and highlighted the challenge of using a new e-learning tool for delivery of noncompulsory training material to health professionals. Reasons for this probably related to the following: (1) the VI was not mandatory learning, so health professionals who did not regularly use vancomycin may not have been motivated to participate; (2) competing education priorities in those health professionals not otherwise intrinsically motivated to participate; (3) lack of time out from clinical responsibilities; (4) the likelihood that multiple staff completed the VI together, meaning that the VI’s reach might have been greater than the results demonstrated; (5) the tool was not targeted toward a specific profession; and (6) not being able to access the VI using hospital computers, which may have hampered widespread use by health professionals during office hours. However, there were only four reports of the VI not loading from 51 survey responses, suggesting that the majority of participants could access the VI. Although the free access website allowed for participation during working hours, there may have been less motivation to perform work-related education in this setting. It was expected that the greatest amount of participation would occur during working hours on hospital computers. Clinical indications for vancomycin were not addressed by the VI, as its primary purpose was to improve knowledge once the decision to prescribe had been made.

Our study presented a model for adopting serious game concepts in combination with humor to develop and conduct Web-based health professional education in a light-hearted, interactive, and entertaining way. This model may be useful in settings where use of face-to-face education is limited by resources and geography. As the VI learning material was made available around the world, it showcased another significant benefit of open Web-based education resources. Health professionals and health care organizations with the same learning needs can reuse the material we have published rather than expending resources to develop similar material.

### Conclusions

We demonstrated a novel Web-based e-learning tool that used humor and some game design principles to deliver health professional education on the commonly used antibiotic vancomycin. The VI was well accepted by users, and it was thus useful for delivering the intended health professional education. Future learning needs for different professional groups were identified through both the Web-based knowledge survey and VI data. This will allow tailoring of face-to-face education programs, in addition to subsequent versions of the VI that will embed robust gaming methodology. Further research will be aimed at measuring the effect on knowledge of the VI compared with a traditional email intervention and examining the impact of the VI on clinical vancomycin use.

## Appendix

Multimedia Appendix 1 Vancomycin Web-based knowledge survey questions.

Multimedia Appendix 2 Vancomycin Interactive questions.

Multimedia Appendix 3 Qualitative survey feedback on the Vancomycin Interactive.

## Abbreviations

IBL: Internet-based learning
IQR: interquartile range
ISLHD: Illawarra Shoalhaven Local Health District
MRSA: methicillin-resistant Staphylococcus aureus
NSW: New South Wales
SESLHD: South Eastern Sydney Local Health District
TDM: therapeutic drug monitoring
VI: Vancomycin Interactive
