# Supplementary material for: Design and Implementation of a Novel Web-Based E-Learning Tool for Education of Health Professionals on the Antibiotic Vancomycin
Source: J Med Internet Res. 2017 Mar 30;19(3):e93. doi: 10.2196/jmir.6971 (PMC5391435; doi:10.2196/jmir.6971)
Supplement: Multimedia Appendix 1 [file jmir_v19i3e93_app1.pdf]

Appendix 1: Vancomycin online knowledge survey questions

1. What is your profession?
2. How much experience do you have with calculating doses of vancomycin?
3. What do you think is the correct loading dose for vancomycin?
4. What is an appropriate maintenance dose for vancomycin for a patient with a creatinine clearance of greater than 90mL/min?
5. How confident are you to determine the administration rate for vancomycin?
6. At what rate should vancomycin be administered to avoid red man syndrome?
7. How confident are you to provide advice on vancomycin monitoring?
8. When should the first level be taken for a dose of 1g 12hrly?
9. What is the usual target range for vancomycin plasma trough levels?
10. Did you refer to any resources to answer these questions?
