# Supplementary material for: Design and Implementation of a Novel Web-Based E-Learning Tool for Education of Health Professionals on the Antibiotic Vancomycin
Source: J Med Internet Res. 2017 Mar 30;19(3):e93. doi: 10.2196/jmir.6971 (PMC5391435; doi:10.2196/jmir.6971)
Supplement: Multimedia Appendix 2 [file jmir_v19i3e93_app2.pdf]

Appendix 2: Vancomycin interactive serious game questions

1. What is an appropriate loading dose for Mrs Jones?
2. What is an appropriate regular dose for Mrs Jones?
3. What fluids should vancomycin be administered in? (more than one correct answer)
4. At what rate do you need to administer the vancomycin?
5. When should the first vancomycin level be taken for a patient receiving 1g 12hrly?
6. What is the target trough level?
7. If the level comes back as 35mg/L what may this mean? More than one answer may apply.
8. The first level comes back as 20mg/L. What should the next dose for this patient be and what is the dose interval?
9. If the level comes back as 26mg/L, what should the next dose and dose interval be for this patient?
10. The course length is likely to be 7 days based on the clinical response of the patient. When should the next level be taken? More than one answer may apply.
