# Supplementary material for: Design and Implementation of a Novel Web-Based E-Learning Tool for Education of Health Professionals on the Antibiotic Vancomycin
Source: J Med Internet Res. 2017 Mar 30;19(3):e93. doi: 10.2196/jmir.6971 (PMC5391435; doi:10.2196/jmir.6971)
Supplement: Multimedia Appendix 3 [file jmir_v19i3e93_app3.pdf]

Appendix 3: Qualitative survey feedback on the vancomycin interactive serious game

**What was good about the vancomycin interactive in comparison to other e-learning modules? (28 responses)**

- could not view
- engaging
- entertaining
- Entertaining
- entertaining easy to understand
- fun and immediate answers available
- Funny, not too much information
- held attention. not just boring power point slides
- i learnt stuff
- Informative and entertaining
- Interesting and memorable (due to jokes and cased based learning)
- It concentrated on vancomycin
- It was an entertaining lesson
- it was engaging & entertaining :-)
- It was fun and had a lighter approach to learning which was very nice! It was still very informative and educational, but the fun nature of Shirley made it more enjoyable to complete.
- It was short and specific for vancomycin
- it was very knowledgable session. it improves my confidence. the main thing is we are familiar with this medication and we quiet often uses at ward.
- It wouldnt work on my work computer
- more real life
- no idea it wouldn't load
- Provides a real world context
- Step by step followed by questions
- Unable to do vancomycin interactive.
- Very entertaining
- Video
- Was interesting and interactive
- Was short and funny

- Was very interesting.....Fun way of learning with the jingle

Did the training provided by the vancomycin interactive meet your needs? If not, what can be improved? (23 responses)

- yes (n=16)
- a few case studies with some monitoring of levels would be useful
- could not view
- Good to be able to print a chart with formulas and normal levels
- I think so, not sure if available but would like a summary of key points that can be printed out at end
- It would be better to have more information before questions e.g. for dosing etc. Or at least have some explanation as to why the questions were wrong.
- no idea it wouldn't load
- not sure
